# 631. The Safety Zone: An Assessment of Home Safety Measures

**DOI:** 10.1093/jbcr/irag033.457

**Published:** 2026-04-06

**Authors:** Shannon L Smith, Miranda L Yelvington, Lori Jordan

**Affiliations:** Arkansas Children's Burn Program, Little Rock, AR; Arkansas Children's Burn Program, Little Rock, AR; Arkansas Children's Burn Program, Little Rock, AR

## Abstract

**Introduction:**

Unintentional injuries in the home environment are a leading cause of preventable burns. According to the 2024 Burn Injury Summary Report, 61% of burns took place at a private residence. For pediatric burns, 58% of cases were related to scalds, most of which occur in the home kitchen or while bathing. Injury prevention requires a comprehensive understanding of current safety practices. Prior research has shown an improvement in adherence to prevention and safety guidelines when education is provided in conjunction with safety equipment. This study reviews reported safety practices of families visiting a family resource center in an academic pediatric hospital.

**Methods:**

Data were collected from the *** Family Resource Center Safety Zone contacts between December 2015 and January 2025. The Safety Zone receives referrals from all outpatient clinics. During this time period, 11.8% of referrals were from the Burn Clinic. This dataset includes safety practices reported during visits to the Safety Zone. Data were captured in REDCap and were self-reported by the parent or caregiver. Queries related to burn injury risk were analyzed for this study to assist with the assessment of the outreach program.

**Results:**

Safety assessments (n = 4228) in English (3696) and Spanish (532) were reviewed to assess home safety practices. For questions related to home fires, 64% reported having a working smoke detector, and 75% reported having a fire escape plan. Kitchen queries revealed that only 18% of families had oven locks, and only 20% had microwaves that children could not open. The majority of families (54.6%) were unaware of the temperature of their water heater, and 67.8% did not check the temperature of their child’s bath water. Safety items and education were provided for areas of need. Commonly distributed items included smoke detectors, oven and microwave locks, bath thermometers, and smoke detectors. In follow-up surveys, 91.4% of respondents reported using items they received from the Safety Zone, 91.4% indicated that the information and education provided made their children safer, and 88.6% reported that the products received helped make their children safer.

**Conclusions:**

Hospital-based outreach services, such as the Safety Zone, provide an opportunity to provide education and prevention materials to patients and families beyond those typically in contact with Burn Outreach services. With burns being a leading cause of pediatric injuries, providing education and materials to all families can be a crucial part of a state-wide prevention strategy.

**Applicability of Research to Practice:**

Partnering with existing resources, such as the Safety Zone, can increase the number of contacts made by Burn Prevention teams and can decrease the risk of burn injury in children who typical outreach measures may not reach.

**Funding for the study:**

N/A.

